# Polymorphism detection of *DGAT1* and *Lep* genes in Anatolian water buffalo (*Bubalus bubalis*) populations in Turkey

**DOI:** 10.5194/aab-65-1-2022

**Published:** 2022-01-03

**Authors:** Raziye Işık, Emel Özkan Ünal, M. İhsan Soysal

**Affiliations:** 1 Faculty of Agriculture, Department of Agricultural Biotechnology, Tekirdağ Namık Kemal University, Tekirdağ 59030, Turkey; 2 Faculty of Agriculture, Department of Animal Science, Tekirdağ Namık Kemal University, Tekirdağ 59030, Turkey

## Abstract

Acyl-CoA: diacylglycerol–acyltransferase 1 (DGAT1) enzyme plays a key role in controlling the synthesis rate triglyceride from diacylglycerol. Leptin (LP, OB, obese) is an important hormone that synthesizes mostly from adipose tissue and regulates glucose metabolism and homeostasis. *DGAT1* and *Lep* genes are closely related to reproduction, growth, milk yield and composition in water buffalo breeds. This study aimed to identify genetic variation in the *DGAT1* and *Lep* gene regions in 150 water buffalo individuals from five different provinces of Turkey using DNA sequencing. A total of 38 nucleotide variations and indels have identified 761 bp long partial intron 2 and exon 3 and 5 ${}^{\prime }$ UTR regions of the *Lep* gene in Anatolian water buffalo populations; 422 bp long partial exon 7–9 and exon 8 regions of *DGAT1* gene were amplified and two mutations were defined in the point of 155 and 275 nucleotide that is three genotypes for S allele and Y allele of *DGAT1* gene in intron 7 in Anatolian buffalo populations, respectively. These SNPs may have an effect on reproduction, growth, milk yield and composition in water buffalo populations and may prove to be useful for water buffalo breeding.

## 1 Introduction

The water buffalo (*Bubalus bubalis*) originated in tropical and subtropical regions such as India, Southeast Asia, and China. Anatolian water buffalo that originated from the Mediterranean subgroup of river buffalo, which is known as the Anatolian water buffalo, adapted to harsh environmental conditions such as low-quality pasture, parasites, and pathogens (Andreas et al., 2010; Soysal, 2013; Konca and Akyüz, 2017).

In Turkey, generally farmers raise a very low number of buffaloes for family consumption in small villages, while big farms with more than 100 heads are located near the big cities. Turkey is composed of seven different geographical regions (Marmara, Aegean, Black Sea, Central Anatolia, Eastern Anatolia, Mediterranean and Southeastern Anatolia regions). Buffaloes are mainly reared in the Marmara Region, Aegean Region, Black Sea Region, Central Anatolia Region, Eastern Anatolia Region and Southeastern Anatolia Region of Turkey (Atasever and Erdem, 2008). The provinces with the highest amount of water buffalo presence are recorded as Samsun, Diyarbakır, Istanbul, Tokat, Bitlis, Mus, Afyon, Kayseri, Sivas and Amasya (FAO, 2021). Buffaloes are substantially raised for milk and meat production in Asian countries, Italy, and Turkey. Buffalo milk that has a high percentage of fat is used for Turkish desserts and making special yogurt. This fatty part of buffalo milk is consumed in the traditional Turkish breakfast called “kaymak”. Moreover, buffalo meat is used in the Turkish sausage-making industry.

The number of water buffaloes in Turkey has increased from 84 000 to 142 000 over the last 10 years (http://www.fao.org/faostat/en/#data/QA,, FAO, 2021) because Anatolian water buffalo are included in a programme for the protection of gene resources by the Ministry of Agriculture and Rural Affairs in Turkey. Also, The National Anatolian Water Buffalo Breeding Project has been carried out with the cooperation of the Republic of Turkey Ministry of Agriculture and Forestry and Anatolian Water Buffalo Breeder Associations since 2013. Thus, some researchers have been handled to identify genetic structure and variation of Anatolian buffalo using ISSR markers (Aytekin et al., 2011), microsatellites (Özkan Ünal et al., 2014; Gargani et al., 2009; Soysal et al., 2007) *DGAT1* gene (Özdil and İlhan, 2012) and leptin gene (Kaplan, 2018a).

Recent research has shown that *DGAT1* and *LEP* genes, where quantitative trait loci are located, are related to reproduction (Demeter et al., 2009; Nasr et al., 2016), growth (Nkrumah et al., 2004, 2005; Kulig and Kmieć, 2009), milk yield and composition (Grisart et al., 2004; Tanpure et al., 2012; Meng et al., 2013) in water buffalo and cattle (Banos et al., 2008). Acyl-CoA: diacylglycerol–acyltransferase 1 (DGAT1) enzyme plays a key role in controlling the synthesis rate triglyceride from diacylglycerol and FA-CoA in adipocyte, which is important to supply the requirement of energy during physiological phases such as pregnancy and lactation. Moreover, *DGAT1* is a functional candidate gene on quantitative trait locus (QTL), which is located on the centromeric end of the bovine chromosome 14 (Bennewitz et al., 2004; Coppieters et al., 1998; Grisart et al., 2002, 2004). In the eighth exon of *DGAT1*, two single-nucleotide polymorphisms (SNPs) lead to the substitution of lysine with alanine (K232A) that is closely related to variation in milk fat content and milk performance (Winter et al., 2002). In some Anatolian, Indian and Chinese water buffalo populations, it was seen that the eighth exon of the *DGAT1* gene has a fixed K allele (Tantia et al., 2006; Shi et al., 2012; Özdil and İlhan, 2012).

Leptin (LP, OB, obese) is an important hormone that synthesizes mostly from adipose tissue and regulates glucose metabolism and homeostasis (Houseknecht et al., 1998; Orrù et al., 2007). It affects carcass traits and body weight, feed intake (Nkrumah et al., 2005; Banos et al., 2008; Kulig and Kmieć, 2009), fertility (Nasr et al., 2016), milk yield and composition (Banos et al., 2008; Nasr et al., 2016), and immune function of cattle and buffalo (Asiamah et al., 2009; Datta et al., 2013). Therefore, the leptin gene is a crucial candidate gene for production traits in buffalo (Nasr et al., 2016). Buffalo leptin gene located on chromosome 8 includes two exons and introns with 167 amino acids (NC_058350) (https://www.ncbi.nlm.nih.gov/nuccore/MF679169, NCBI, 2021). Nasr et al. (2016) revealed the associations with polymorphism in the second exon region of leptin gene and fertility, as well as production traits in Egyptian buffaloes.

The aim of this study was to identify genetic variation in the *DGAT1* and *Lep* gene regions in 150 water buffalo individuals from five different provinces of Turkey using DNA sequencing.

## 2 Materials and methods

### 2.1 Experimental animals and blood collection

**Figure 1:**
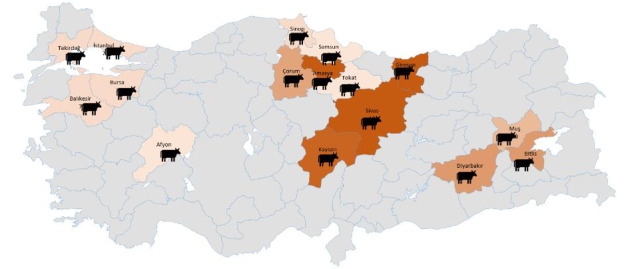
Location of the Anatolian water buffalo samples collected in this study.

All procedures involving manipulation of the animals were conducted in accordance with Turkish Guidelines on Animal Welfare. This research was approved by the Ethics Committee on the Use of Animals, Republic of Turkey Ministry of Agriculture and Forestry, Institute of Pendik Veterinary Control Ethics Committee (case no. 2013/12). In this study, the 150 unrelated Anatolian water buffalo blood samples used (0–6 months, 1–3 years; males, n=46 ; females, n=104 ) were collected from East Anatolia (Bitlis, Diyarbakır, Muş, n=40 ), Black Sea (Amasya-Merzifon, Samsun, Tokat, Çorum, Giresun, Sinop, n=50 ), Central Anatolia (Kayseri, Sivas, n=17 ), and Aegean–Marmara (Afyon, Istanbul-Nakkaş/Danamandıra, Balıkesir, Bursa, Tekirdağ-Saray, n=43 ) regions of Turkey (Fig. 1). Blood samples from animals were collected in 5 mL vacuum tubes, including EDTA as an anticoagulant, and stored at - 80 ${}^{\circ }$ C till the DNA extraction. Genomic DNA was isolated by using a commercial DNA isolation kit (GeneJET Whole Blood Genomic DNA Purification Mini Kit, Thermo Fisher Scientific) according to the manufacturer's instructions.

### 2.2 Primers, total DNA extraction and PCR

Primer sequences of *DGAT1* and *Lep* genes that are used for amplification and sequencing are shown in Table 1. Reverse primer of *Lep* gene is designed using Primer3web online software (version 4.1.0) (https://primer3.ut.ee/, last access: 12 March 2021) based on NCBI Reference Sequence NC_058350 (91132528-91149368). For PCR amplification reactions, the 35 µ L PCR volume contained 20 ng genomic DNA, 0.5 µ M of each primer, 1× PCR buffer ((NH ${}_{4}$ ) ${}_{2}$ SO ${}_{4}$ ), 200 µ M dNTP, 1.5 mM MgCl ${}_{2}$ and 1 U of Taq DNA polymerase (Taq DNA Polymerase, Thermo Scientific, USA). The cycling protocol was 4 min at 94 ${}^{\circ }$ C for initial denaturation, 35 cycles of amplification; 94 ${}^{\circ }$ C for 30 s, 56.1 ${}^{\circ }$ C annealing for 30 s, 72 ${}^{\circ }$ C for 2 min and 15 min at 72 ${}^{\circ }$ C for final extension. Afterward, the PCR products were checked on 1 % agarose gel using horizontal electrophoresis and the gels were stained using SafeView™ Classic (Applied Biological Material Inc. Canada).

**Table 1:** Primer sequences of *DGAT1* and *Lep* genes.

Gene regions	Primers	Length (bp)	References
*DGAT1*	F: 5 ${}^{\prime }$ -CAC CAT CCT CTT CCT CAA G-3 ${}^{\prime }$ R: 5 ${}^{\prime }$ -GGA AGC GCT TTC GGA TG-3 ${}^{\prime }$	412	Shi et al. (2012)
*Lep*	F: 5 ${}^{\prime }$ -GTC TGG AGG CAA AGG GCA GAT-3 ${}^{\prime }$ R: 5 ${}^{\prime }$ -GAT GGT CCA AAG GCT CTG AA-3 ${}^{\prime }$	758	Lien et al. (1997) (forward primer)

### 2.3 Sequencing and data analysis

A total of 412 bp of *DGAT1* gene and 558 bp of *Lep* genes were sequenced on an Applied Biosystems 3500XL Genetic Analyzer System (Applied Biosystems, USA) to determine those gene sequences. *DGAT1* and *Lep* fragments were blasted with the NCBI BLAST tool (https://blast.ncbi.nlm.nih.gov/Blast.cgi, last access: 12 March 2021) to defined orthologue sequences on the NCBI GeneBank database. Homolog sequences were downloaded by NCBI GenBank database and aligned with ChromasPro 2.4.3 program (Technelysium Pty Ltd) and BioEdit (v7.0.9) Sequence Alignment Editor with Clustal W multiple alignment modules (Hall, 2011). A heatmap was performed for the differential nucleotide (SNP) of the selected sequences of *Lep* gene region using an R script with the R function “heatmap” of the package “ggplot2” based on the genetic distance of sequences analysed with the MEGA7 software (Molecular Evolutionary Genetics Analysis, version 6.0). The 3D tertiary structure of the proteins based on the studied *Lep* gene region was predicted using the I-TASSER server (Yang et al., 2015). The genotypic and allelic frequencies of the Lep gene and the Hardy–Weinberg equilibrium of the populations were calculated using the PopGene program (Yeh et al., 2000).

## 3 Results

**Figure 2:**
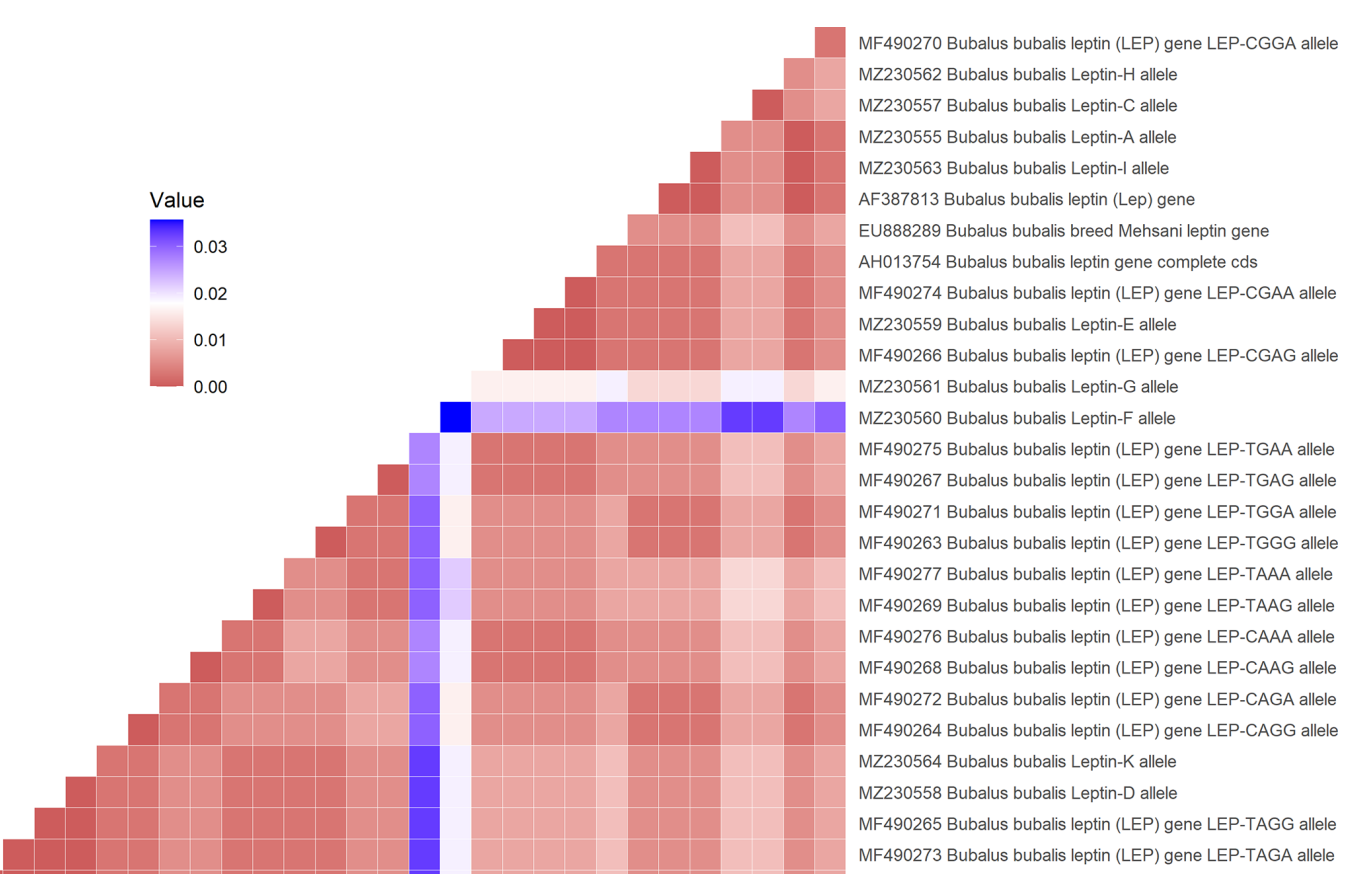
Heatmap showing the differential nucleotide (SNP) of the selected sequences of *Lep* gene region.

In the studied samples, the partially second intron and third exon regions of the *leptin* gene were amplified. A total of 758 and 761 bp length samples were sequenced (AH013754). These variations were deposited in the NCBI GenBank database with the accession numbers MZ230555–MZ230565. As 18 nucleotide changes in the third exon and 20 nucleotide changes in the intron regions, a total of 38 point nucleotide variations have been identified in the studied regions of the *Lep* gene (Table S1 in the Supplement). Also, 3 bp insertion/deletions (indels) have defined position 47–49 (position 3998 at AH013754) in the second intron region of the *Lep* gene for the first time. In this study, we have detected three nonsynonymous amino acid changes and 13 silent mutations in third exon sequence of *Lep* amino acid residues in Anatolian buffalo breeds (Table 2). The amino acids change as T39A and V47I have been defined for the first time in Anatolian buffalo breeds. According to Fig. 2, the heatmap revealed the differential nucleotide (SNP) of the *Lep* gene region based on the genetic distance of sequences. While *Lep* A, C, and H alleles were close to MF490270 *LEP*-CGGA allele, *Lep*-I allele was close to HE605297 sequence, which was retrieved from the NCBI GenBank database. The *Lep*-B allele of Anatolian buffalo was the same clade as the *LEP*-TAGA allele (MF490273).

**Table 2:** The nucleotide variations and amino acid changes in *Lep* amino acid residues.

Location ${}^{*}$	Codon change	Amino acid	Mutation type
31	TTA > TTG	Leucine (L)	Silent
32	GCG > GCA	Alanine (A)	Silent
39	ACC > GCC	Threonine(T) > alanine (A)	Missense
47	GTC > ATC	Valine (V) > isoleucine(I)	Missense
50	TCT > TCC	Serine (S)	Silent
71	TTG > TTA	Leucine (L)	Silent
72	CCG > CCA	Proline (P)	Silent
77	CTG > CTA	Leucine (L)	Silent
79	AGT > AGC	Serine (S)	Silent
83	TTG > TTC	Leucine (L)	Silent
84	GGC > GGT	Glycine (G)	Silent
85	GTC > GTT	Valine (V)	Silent
102	CTG > CTA	Leucine (L)	Silent
105	TCA > TCG	Serine (S)	Silent
111	CGG > CAG	Arginine (R) > glutamine (Q)	Missense
116	AGT > AGC	Serine (S)	Silent
117	CCT > CCC	Proline (P)	Silent

${}^{*}$ Location is the point of mutations that occurred in the studied *Lep* gene amino acid residues.

**Figure 3:**
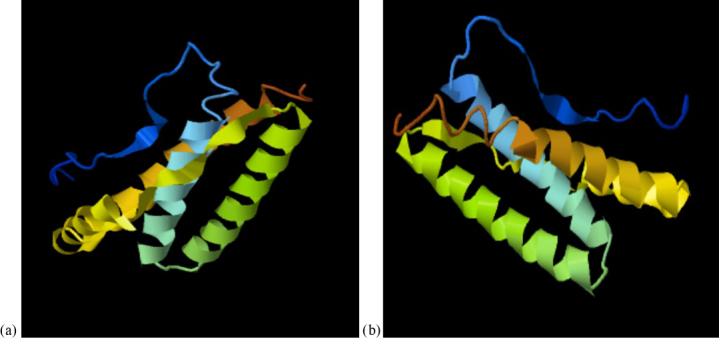
The predicted 3D tertiary structure of the Anatolian water buffalo leptin peptide; **(a)** native structure and **(b)** the effect of the detected amino acid changes on its 3D tertiary structure.

The predicted 3D tertiary structure of the Anatolian water buffalo leptin peptide and the effect of the detected mutations on its 3D tertiary structure are shown in Fig. 3. While the C-score of native 3D tertiary structure of leptin peptide was -0.74 (estimated TM-score = 0.62±0.14 , estimated RMSD = 5.8±3.6 Å), the C-score of leptin peptide with the three mutations was -0.69 (estimated TM-score = 0.63±0.14 , estimated RMSD = 5.7±3.6 Å).

The genotype and allele frequencies belong to some SNPs of *Lep* gene are listed in Table 3. *Lep*-G allele frequencies were 0.85, 0.83 and 0.82 for the point of 164, 287 and 403 SNPs, respectively. While *Lep*-C allele frequency was 0.67, *Lep*-T allele frequency was 0.33 for the point of 562 SNP. In this study, the genotype distributions in SNPs of *Lep* gene were not found in Hardy–Weinberg equilibrium ( p<0.05 ) except for SNP in the point of 287 ( p>0.05 ).

**Table 3:** The genotype and allele frequency belong to some SNPs of the *Lep* gene in Anatolian water buffaloes.

SNP position	N	Genotype			Allele		$\chi^{2}$
		frequencies			frequencies		
		AA	GG	AG	A	G	
164	150	0.07 (10)	0.76 (114)	0.17 (26)	0.15	0.85	16.57
287	150	0.09 (8)	0.74 (108)	0.17 (34)	0.17	0.83	5.07
403	150	0.05 (7)	0.68 (102)	0.27 (41)	0.18	0.82	1.14 ${}^{*}$
		TT	CC	TC	T	C	
562	150	0.17 (25)	0.51 (77)	0.32 (48)	0.33	0.67	11.14

${}^{*}$ $\chi^{2}$ Hardy–Weinberg equilibrium (HWE).

**Figure 4:**
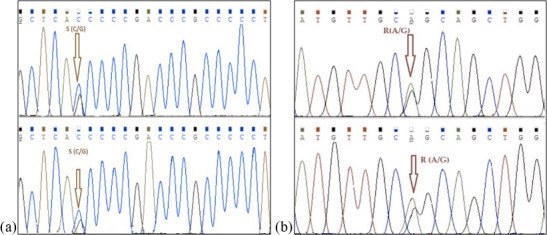
Nucleotide changes from C to G in intron 7 region of *DGAT1* gene (point of 155 nucleotide, **a**) and from A to G in point of 403 nucleotide *Lep* gene (point of 111 amino acid, **b**).

Mediterranean water buffalo *DGAT1* gene consists of 17 exons located on chromosome 15, and it is 10.224 bp long in the NCBI reference sequence (NC_037559). A total of 422 bp long partial exon 7–9 and exon 8 regions of *DGAT1* gene in studied Anatolian buffalo populations (NC_037559) are amplified. A mutation is detected in the point of 155 nucleotide that is homozygote C and G and heterozygote S allele of *DGAT*1 gene in intron 7. The nucleotide changes from C to G in the *DGAT1* gene and from A to G of *Lep* gene as shown in Fig. 4. Also, we identified a variation at the point of 275 nucleotide that is homozygote C and T and heterozygote Y allele of *DGAT1* gene in intron 7. These variations are deposited in the NCBI GenBank database with the accession numbers MZ230551-MZ230554. Table 4 shows the nucleotide differences of reference bovine and buffalo sequences that deposited in the NCBI GenBank and studied sample sequences in the current research.

**Table 4:** The nucleotide variations between the studied sequences and reference sequences of *DGAT*1 gene.

Location	Nucleotide change	Anatolian buffalo	References ${}^{*}$	
22	T	C	AM263422	Bovine (*Bos taurus*)
43	T	C	JQ627611	Buffalo
94	G	C	FJ014705	Bovine
104	A	C	JQ897351	Bovine (*Bos taurus*)
130, 131	TC	GA	MF351623	Bovine (*Bos indicus*)
154	G	A	JF894305	Buffalo
155	C	C, G, S	MF490259	Buffalo
171	A	T	DQ408185	Bovine (*Bos indicus*)
201, 202	GC	KK	JQ897352	Bovine (*Bos taurus*)
201, 202	GC	AA	JQ897353	Bovine (*Bos taurus*)
228	G	A	AM263425	Bovine (*Bos taurus*)
243	G	A	DQ435287	Bovine (*Bos indicus*)
275	C	C, T, Y	JF894305	Buffalo
289	A	G	AY999090	Buffalo
296	T	G	FJ014705	Buffalo
303	C	T	AM263423	Bovine (*Bos taurus*)
357	C	G	JQ627612	Buffalo
362	A	T	DQ435287	Bovine (*Bos indicus*)
365	T	G	AM263424	Bovine (*Bos taurus*)
373	G	C	JQ627610	Buffalo

${}^{*}$ The reference sequences were deposited by previous studies to the NCBI GenBank.

## 4 Discussions

In this study, the partially second intron and third exon regions of the leptin gene and the partial exon 7, 9 and exon 8 regions of *DGAT1* gene were amplified in Anatolian buffalo populations. A total of 38 nucleotide variations in the *Lep* gene and 4 nucleotide variations in *DGAT1* gene have been identified in the studied populations.

Nasr et al. (2016) reported three genotypes (AA, AG and GG) of leptin locus, and the animals that have AA genotype had the highest milk yield (2332.34 kg, p=0.04 ) and fat yield (155.75 kg, p=0.06 ) in Egyptian buffaloes. But they revealed that the GG genotype (43.67 kg, p=0.02 ) had higher birthweight compared with AA genotype (39.35 kg, p=0.02 ). Orrù et al. (2007) reported that the G allele (0.640) was predominantly in the T1131G locus of leptin gene in Italian and Egyptian buffaloes. Kaplan (2018b) studied the same region of the *Lep* gene and reported three genotypes as TT, GT and GG in a small population of Anatolian buffaloes. In Italian and Egyptian buffalo, Orrù et al. (2007) reported 11 SNPs: G3333A, C1221T, G3195A, C1221T, G3434A, T1015C, C1071T, G1072A, T1081C, T1143C and T1145G loci of *Lep* gene. Jhala et al. (2009) identified three variations in exon 3 of leptin gene as A42G, A44G and A250G in Mehsana buffalo. In another research on this buffalo breed carried out by Tanpure et al. (2012), five SNPs were determined at positions 98, 111, 172, 209 and 266 in the first intron of leptin gene. In Mediterranean water buffaloes, Scatà et al. (2012) revealed eight SNPs (A83G, A90G, A121G, G256T, A283G, G959T, A1010C, G1254A) in the 5 ${}^{\prime }$ flanking and first exon regions of leptin gene. The non-synonymous SNPs at positions A114G (EU888289.1), C163A (EU825672.1), A211G (JQ045625.1), G288A (AY177609.1), A310G (KP864436.1), A322G (JN689387.1), G330C (JQ045625.1), C348T (JQ045625.1), T360C (HE605297.1) and G379A (Orru et al., 2007) caused amino acid changes (S71G, T87N, N103S, E129K, E136G, Y140C, E143Q, R149W, S153P, and R159Q, respectively) in LEP polypeptide. The T27C mutation (KP897166.1), which is close to the 3 ${}^{\prime }$ splicing site of the second intron region of the *Lep* gene, dislocates a splicing silencer and creates a new intron splicing silencer, while A114G and A310G SNPs of the third exon dislocate two splicing enhancers and create new exonic splicing enhancers that may cause alternative splicing and intron retention (Braunschweig et al., 2014; Mahrous et al., 2020).

*Lep*-G allele frequencies were high for the point of 164, 287 and 403 SNPs, respectively. The genotype distributions in SNPs of *Lep* gene were not found in Hardy–Weinberg equilibrium mostly ( p<0.05 ). This can be because of adaptation or fitness, which is a result of random effects of genetic drift in evolutionary process (Andrews, 2010).

In this study, the C-score of the native 3D tertiary structure of leptin peptide was -0.74 , the C-score with the three amino acid changes T39A, V47I and R111Q of leptin peptide was -0.69 . Mahrous et al. (2020) revealed that while the S71G improved the stability of the leptin protein, R159Q decreased the stability of mature leptin peptide tertiary structure -0.25 kcal/mol. Also, Mahrous et al. (2020) reported that the amino acid N103S in leptin polypeptide is conserved in humans and river buffalo, but this amino acid variation does not exist in Anatolian water buffalo populations.

Li et al. (2018) revealed an SNP (g.9046T > C) caused a nonsynonymous amino acid change from arginine to histidine in exon 17 of *DGAT1* gene, which was significantly associated with the fat percentage that TT genotype had a significantly higher fat percentage than the CC genotype in Riverine buffalo and Swamp buffalo. Similar to Li et al. (2018), de Freitas et al. (2016) carried out the nonsynonymous SNP g.11785 T > C that causes the alteration of alanine to valine (Ala484Val) in exon 17 of the *DGAT1* gene that was significantly related to fat and protein percentage of milk in Murrah buffaloes in Brazil ( P<0.05 ) (de Freitas et al., 2016). Contrary to de Freitas et al. (2016), Meng et al. (2013) found that the SNP c.1350C > G in exon 17 was not related to milk yield and compositions in Dehong and hybrid (F1: Mora buffalo × Dehong buffalo) buffaloes. In this study, the amino acid change from alanine to valine was not detected in exon 17 of the *DGAT1* gene, since strict breeding programmes were not implemented for milk yield and composition in Anatolian buffaloes. In Murrah buffalo breed, Cardoso et al. (2015) reported that *DGAT1*-VNTR (variable number of tandem repeats) in the promoter region was significant for the fat percentage of milk because of a located binding site of Sp1 (specificity protein 1), which is a transcription factor for the mRNA synthesis.

Cesarani et al. (2021) revealed that WssGBLUP is a weighted version of ssGBLUP, which is some major genes or SNPs, as *DGAT1* for fat content has more weights that could increase the accuracy of breeding values for growth, milk traits.

## 5 Conclusion

Many studies have been carried out about relations between *DGAT1* and *Lep* genes and economic production traits. In this study, 38 polymorphisms, indels in the *Lep* gene region and several variations in the *DGAT1* gene region were identified which will improve reproduction, growth, milk yield and composition traits in water buffalo based on marker-assisted selection. For this reason, in future studies, these relationships should be defined in water buffalo populations.

## Supplement

10.5194/aab-65-1-2022-supplement The supplement related to this article is available online at: https://doi.org/10.5194/aab-65-1-2022-supplement.

## Data Availability

The data are available from the corresponding author upon request.
