# Supplementary material for: Polymorphism detection of DGAT1 and Lep genes in Anatolian water buffalo (Bubalus bubalis) populations in Turkey
Source: Arch Anim Breed. 2022 Jan 3;65(1):1–9. doi: 10.5194/aab-65-1-2022 (PMC8738919; doi:10.5194/aab-65-1-2022)
Supplement: The supplement related to this article is available online at: https://doi.org/10.5194/aab-65-1-2022-supplement. [file aab-65-1-supplement.pdf]

Supplement of Arch. Anim. Breed., 65, 1–9, 2022
https://doi.org/10.5194/aab-65-1-2022-supplement
© Author(s) 2022. CC BY 4.0 License.

Open Access
Archives Animal Breeding

Supplement of

Polymorphism detection of *DGAT1* and *Lep* genes in Anatolian water buffalo (*Bubalus bubalis*) populations in Turkey

Raziye Işık et al.

Correspondence to: Raziye Işık (risik@nku.edu.tr) and Emel Özkan Ünal (ozemel@nku.edu.tr)

The copyright of individual parts of the supplement might differ from the article licence.

Location	Nucleotide change	NCBI Accession Number	Sex	Age
46	C/G	MZ230555	Female	3
51	T/C	MZ230555	Male	6
52	C/G	MZ230556	Male	12
56	T/C	MZ230556	Female	15
164	A/G/R	MZ230557	Female	3
167	G/A	MZ230557	Male	30
186	A/G	MZ230558	Male	18
210	G/A	MZ230558	Female	34
221	T/C	MZ230559	Female	19
284	G/A	MZ230559	Female	4
287	G/A/R	MZ230560	Female	28
302	G/A	MZ230558	Male	23
308	T/C	MZ230561	Female	6
318	T/C	MZ230560	Female	32
323	C/Y	MZ230562	Female	12
326	C/Y	MZ230556	Male	16
338	C/Y	MZ230563	Female	21
377	G/A	MZ230564	Male	3
386	A/T	MZ230557	Female	24
403	G/A/R	MZ230559	Male	15
419	T/C	MZ230561	Male	33
422	T/Y	MZ230555	Female	4
489	C/T	MZ230562	Male	20
490	A/G	MZ230557	Female	11
504	C/Y	MZ230563	Male	3
549	C/T	MZ230556	Female	12
562	T/C/Y	MZ230565	Female	10
586	C/T	MZ230558	Female	6
587	G/R	MZ230561	Female	17
616	A/G	MZ230564	Female	26
622	C/T	MZ230562	Male	3
630	A/G	MZ230559	Female	22
656	G/C	MZ230560	Male	12
662	C/T	MZ230563	Male	25
663	G/R	MZ230555	Female	4
678	A/G	MZ230565	Male	20
694	C/G	MZ230560	Female	26
707	C/G	MZ230558	Female	14
